# Common and rare variant analyses implicate late-infancy cerebellar development and immune genes in ADHD

**DOI:** 10.1186/s11689-025-09626-4

**Published:** 2025-06-20

**Authors:** Yuanxin Zhong, Larry W. Baum, Justin D. Tubbs, Rui Ye, Lu Hua Chen, Tian Wu, Se-Fong Hung, Chun-Pan Tang, Ting-Pong Ho, Robert Moyzis, James Swanson, Chi-Chiu Lee, Pak C. Sham, Patrick W. L. Leung

**Affiliations:** 1 Department of Psychiatry, Li Ka Shing Faculty of Medicine, The University of Hong Kong, Hong Kong SAR, China; 2 State Key Laboratory of Brain and Cognitive Sciences, The University of Hong Kong, Hong Kong SAR, China; 3 Psychiatric and Neurodevelopmental Genetics Unit, Center for Genomic Medicine, Massachusetts General Hospital, Boston, MA USA; 4 Department of Psychiatry, Harvard Medical School, Boston, MA USA; 5 Stanley Center for Psychiatric Research, Broad Institute of MIT and Harvard, Cambridge, MA USA; 6 Department of Rehabilitation Sciences, Faculty of Health and Social Sciences, The Hong Kong Polytechnic University, Hong Kong SAR, China; 7 Department of Mathematics, Hong Kong Baptist University, Hong Kong SAR, China; 8 Department of Psychiatry, The Chinese University of Hong Kong, Hong Kong SAR, China; 9 Kwai Chung Hospital, Hong Kong SAR, China; 10 Department of Psychology, The Chinese University of Hong Kong, Hong Kong SAR, China; 11 Department of Biological Chemistry, University of California, Irvine, Irvine, CA USA; 12 Department of Pediatrics, University of California, Irvine, Irvine, CA USA; 13 Centre for PanorOmic Sciences, The University of Hong Kong, Hong Kong SAR, China

**Keywords:** ADHD, Common variant, Low-frequency / rare variant, Cerebellum, Late-infancy, Immune response

## Abstract

**Objective:**

Attention-deficit hyperactivity disorder (ADHD) is a common neuropsychiatric disorder with a significant genetic component. The latest genome-wide association study (GWAS) meta-analysis of ADHD identified 27 whole-genome significant risk loci in the European population. However, genetic risk factors for ADHD are less well-characterized in the Asian population, especially for low-frequency / rare variants.

**Methods:**

In this study, we aimed to investigate the contributions of both common and low-frequency / rare variants to ADHD in a Hong Kong sample. Our sample comprised 279 cases and 432 controls who underwent genotyping using the Illumina Infinium Global Screening Array. We employed various analytical methods at different levels, while also leveraging multi-omics data and large-scale summary statistics to comprehensively analyze the genetic basis of ADHD.

**Results:**

We identified 41 potential genomic risk loci with a suggestive association (*p* < 1e^−4^), pointing to 111 candidate risk genes, which were enriched for genes differentially expressed during late infancy brain development. Furthermore, tissue enrichment analysis implicated the involvement of the cerebellum. At the polygenic level, we also discovered a strong genetic correlation with resting-state functional MRI connectivity of the cerebellum involved in the attention/central executive and subcortical-cerebellum networks. In addition, an accumulation of ADHD common-variant risks found in European ancestry samples was found to be significantly associated with ADHD in the current study. In low-frequency / rare variant analyses, we discovered the correlations between ADHD and collapsing effects of rare damaging variants in *TEP1*, *MTMR10*, *DBH*, *TBCC*, and *ANO1*. Based on biological and functional profiles of the potential risk genes and gene sets, both common and low-frequency / rare variant analyses demonstrated that ADHD genetic risk was associated with immune processes.

**Conclusions:**

These findings re-validate the abnormal development of the neural system in ADHD and extend the existing neuro-dysfunction hypothesis to a multi-system perspective. The current study identified convergent risk factors from common and low-frequency / rare variants, which implicates vulnerability in late-infancy brain development, affecting especially the cerebellum, and the involvement of immune processes.

**Supplementary Information:**

The online version contains supplementary material available at 10.1186/s11689-025-09626-4.

## Introduction

Attention-deficit/hyperactivity disorder (ADHD) is a neurodevelopmental disorder that has a relatively high prevalence worldwide, around 5% in children and 2.5% in adults [1]. In Hong Kong, one in every 15 children has ADHD [2]. It is characterized by inattention, hyperactivity and impulsiveness, with downstream long-term effects on school and work performance, domestic and social relationships, and mental health [3–6], causing a considerable burden on the family and society [7–9].

Genetic factors contribute to ADHD, as shown by twin heritability estimates of up to 0.74 [10]. The latest large-scale genome-wide association study (GWAS) meta-analysis of ADHD, comprising 38,691 individuals with ADHD and 186,843 controls, revealed 27 genome-wide significant risk loci and highlighted 76 candidate risk genes, which showed enrichment among genes expressed in early brain development [11]. This study not only provided deeper insight into the genetic structure of ADHD, but also demonstrated the importance of common variants in ADHD risk accounting for 14–22% of the phenotypic variation [11], consistent with the estimates from previous studies [12]. However, all cohorts included in this meta-analysis are of European ancestry [11]. A previous GWAS with 1,040 ADHD cases and 963 controls, recruited from a homogeneous Han Chinese sample, demonstrated a statistically significant (*p* = 0.0072) but modest (r_g_ = 0.39) correlation in SNP effect sizes with those estimated from the European Ancestry ADHD samples [13]. These results suggest that there may be substantial heterogeneity in the effects of common genetic variants across Asian and European ancestries, so that larger studies in Asian populations are necessary for understanding their biological mechanisms and producing more accurate risk predictions.

Rare variants, which can contribute up to one-fourth of heritability [12, 14], are another major genetic contributor to ADHD. ADHD cases display a higher load of rare protein-truncating variants (rPTVs) in genes with high genetic constraint scores [15, 16]. A large-scale European-ancestry study suggests a convergence of genes containing common and rare risk variants, by showing that ADHD cases have an excess burden of rPTVs in genes identified from common variant analyses [11]. However, due to methodological limitations and the higher cost of whole genome or whole exome sequencing, the analyses of rare variant contributions to ADHD are still in their early stage.

Compared to studies conducted in European populations, ADHD genetic studies in Chinese are still limited in sample size [13, 17, 18]. To better understand the genetic architecture underpinning ADHD in the Chinese population, we tested the contribution of common variants and the burden of rare deleterious variants with samples recruited in Hong Kong. We integrated multi-omics data to identify potentially causal genes and gain biological insights. We characterized the polygenic architecture of ADHD and its overlap with other phenotypes by polygenic score (PGS) analyses.

## Methods

**Fig. 1:**
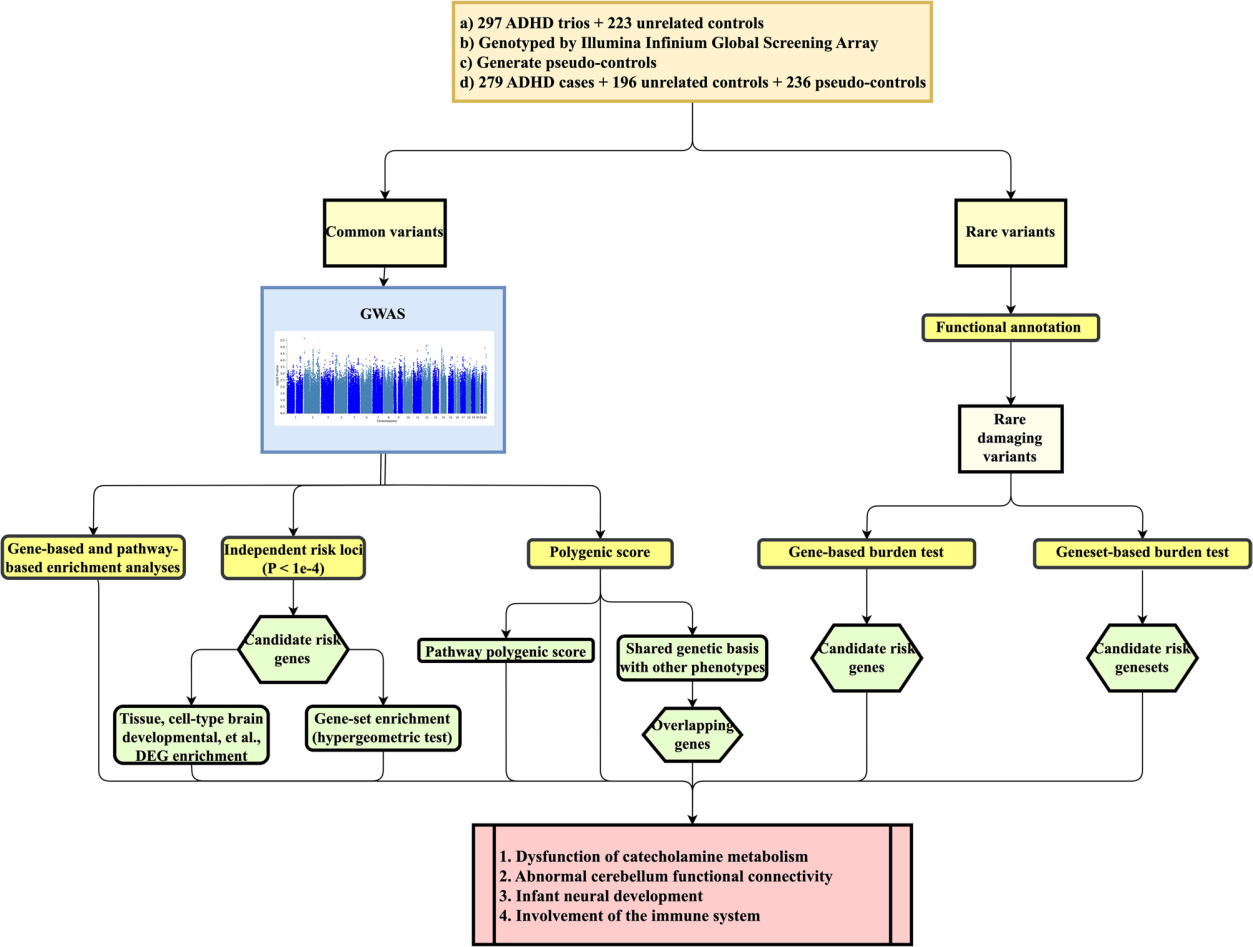
An overview of the current study design. DEG, differentially expressed gene

Figure 1 shows an overview of the current study design.

### Samples

Participants in this study consisted of 297 boys aged 6–11 years with a primary clinical diagnosis of ADHD (including 254 family trios comprising a boy with ADHD and his two biological parents), and 223 boys without ADHD who served as age- and gender-matched typically-developing controls. Additional inclusion criteria were Chinese ethnicity and studying in local mainstream primary schools. Exclusion criteria for the probands were IQ below 80, psychosis, bipolar disorder, Tourette’s syndrome, chronic motor or vocal tic disorders, or serious neurological or medical conditions. For the controls, the parents were asked a set of screening questions about any behavioural and emotional problems of their children. Children whose parents reported concerns, including any symptoms of ADHD, ASD, or other particular learning issues, or who had received complaints or assessment referrals on these issues from schools or other parties, or who were diagnosed with a psychiatric disorder, were excluded from participation as controls. The probands were recruited from child and adolescent psychiatric services of three local public hospitals, while the controls were recruited from mainstream schools and advertisements in social media.

The Joint Chinese University of Hong Kong-New Territories East Cluster, the Hospital Authority Kowloon Central and Kowloon West Cluster Clinical Research Ethics Committee provided ethical approval for this study, which complies with the most recent Declaration of Helsinki. All participants provided informed consent for the current study. Further details on the constitution and recruitment of the samples can be found from a previous publication [19].

### Genotyping, quality control and imputation

Genotyping was performed using the Illumina Infinium Global Screening Array (GSA), whose backbone comprises common and low-frequency / rare variants selected according to research value. Pre-imputation quality control (QC) was performed using established protocols [20, 21]. Briefly, we eliminated subjects and SNPs in the analysis according to the following quality control criteria: SNP call rate < 0.98, subject call rate < 0.98, autosomal heterozygosity deviation (|F_het_| > 0.2), difference in SNP missingness rate between cases and controls > 0.02, and deviation from SNP Hardy-Weinberg equilibrium (HWE) (*p* < 1e^−6^). Variants with minor allele frequency (MAF) in full, case-only, and control-only samples significantly deviating (*p* < 1e^−3^) from that in the 1000 Genomes Project phase 3 data of the East Asian population [22] were also excluded. Based on principal component analysis projecting to the 1,000 Genomes Project phase 3 data of the East Asian population, we removed genetic outliers. For trio data, we generated pseudo-controls from phased haplotypes before imputation. Due to the design features of the GSA, we separated the common (MAF > 0.05) and low-frequency / rare variants according to the above external reference panel (Figure S1).

Imputation of common variants was performed based on 1000 Genomes Project phase 3 data of the East Asian population [22] using the Michigan Imputation Server [23]. Variants with imputation INFO score > 0.8 and MAF > 0.05 were included for further analysis of common variants. There were 4,111,958 common variants included after quality control. For low-frequency / rare variants, imputation was not attempted, and we used posterior probabilities (GP) index > 0.8 in the VCF (Variant Call Format) file to ensure the relatively high quality of the variants. Functional annotation was conducted by Variant Effect Predictor (VEP) [24] based on all available implemented data. Only variants with MAF < 0.05 in the 1000 Genomes Project phase 3 data of East Asians [22] or in gnomAD [25], and categorized as having medium or high functional impact by Ensembl, were included in subsequent low-frequency / rare variant analyses. A total of 7,047 low-frequency / rare variants were included.

### Common variant GWAS

GWAS was carried out with the imputed additive genotypes using logistic regression of ADHD cases versus unrelated controls and pseudo-controls in PLINK 1.9 (www.cog-genomics.org/plink/1.9/) [26]. To correct for population stratification, the top five principal components were added as covariates. A two-sided genome-wide significant *P*-value threshold of 5e^−8^ and a suggestive *P*-value threshold of 1e^−4^ [11, 13] were adopted to allow for multiple testing.

### Gene-based association and gene-set analysis

Gene-based association analysis and gene-set analysis were conducted by MAGMA v.1.08 with default settings [27]. Gene-based analysis was performed for 19,093 protein-coding genes and we considered the 21 candidate risk genes reported in a previous ADHD GWAS analysis conducted using Chinese Han samples [13] separately. For gene-set analysis, testing was done on curated gene sets and Gene Ontology (GO) terms (*N* = 15,485) from MsigDB [27, 28]. Five principal components were used as covariates. We used a Bonferroni correction to adjust for multiple testing. Accounting for the number of terms (gene/gene-set) examined, thresholds for genome-wide significance were determined (*p* = 0.05/19,093 = 2.62 e^−6^ for gene-based association analysis, *p* = 0.05/21 = 0.0024 for candidate gene-based association analysis and *p* = 0.05/15,485 = 3.23e^−6^ for gene-set analysis).

### Tissue-specific and cell type-specific gene expression analysis

Correlations between ADHD genome-wide gene-based associations and gene expression patterns in specific tissues were investigated by MAGMA Tissue Expression analysis implemented in the FUMA (Functional Mapping and Annotation) GWAS platform [29] with expression data from GTEx (54 tissue types) and BrainSpan (brain samples at 29 different ages). Cell type-specific expression analysis was performed by FUMA’s CellType model with 13 single-cell RNA-sequencing datasets from the human brain, the same as those used in a recent ADHD meta-analysis [11].

### Mapping risk variants to genes and enrichment analyses

In addition to gene-based tests on previously reported candidate genes, we also performed further analysis to link risk variants to their potentially novel target genes. We input the GWAS results into FUMA [29], with pre-defined top-lead SNPs being identified as those with *p*-values below the suggestive threshold after clumping by PLINK (www.cog-genomics.org/plink/1.9/) [26] (r^2^ < 0.1). For each pre-defined top-lead SNP, other SNPs in high linkage disequilibrium (LD) (r^2^ > 0.8) with it were regarded as candidate causal variants that constitute an independent association signal. Then, genomic risk loci were defined from these independent signals by merging those separated by less than 250 kilobases.

In each locus, candidate causal variants were connected to genes based on genomic location, eQTL (expression quantitative trait loci) data, and chromatin interaction mapping data in human brain tissues and blood samples (see Supporting Information), as implemented in FUMA under the default settings [29]. The mapped candidate risk genes were utilized in further gene-set enrichment analyses by FUMA’s GENE2FUNC module to determine whether they were enriched among (1) genes differentially expressed in specific tissues derived from GTEx [30] (54 tissue types), (2) genes differentially expressed at specific brain developmental stages derived from BrainSpan [31, 32] (brain samples from individuals at 29 different ages and at 11 general developmental stages), and (3) genes encoding proteins in specific pre-defined gene sets implemented in GENE2FUNC. DEGs were identified through a differential expression analysis comparing the target tissue/time point against the remaining data correspondingly. A hypergeometric test was used to determine the statistical significance of the enrichment of ADHD candidate risk genes against all protein-coding genes as background genes. Bonferroni correction was applied to adjust for the multiple testing.

### Polygenic score analysis

We used PRSice2 [33] to perform polygenic score (PGS) analysis with *p*-value thresholds (5e^−8^, 1e^−7^, 1e^−6^, 1e^−5^, 1e^−4^, 1e^−3^, 0.01, 0.02, 0.03, 0.04, 0.05, 0.1, 0.2, 0.3, 0.4, 0.5, 1) and an external European LD reference panel generated from the 1000 Genomes Project phase 3 data [22]. Although using a trans-ancestry PGS may lead to reduced prediction accuracy, we leveraged summary statistics from European ancestry in PGS calculation for the following reasons, (1) summary statistics from ADHD GWAS in Chinese samples are limited by sample size or are not publicly available; (2) studies in European population have larger sample sizes, thus provide better statistical power for prediction; and (3) Wang, Guo [34] found that the causal variants identified in European ancestry GWAS are common across continents. The latest publicly available Psychiatric Genomics Consortium (PGC) summary statistics of five major psychiatric disorders [i.e., ADHD [11], autism spectrum disorder [35], bipolar disorder [36], major depressive disorder [37], and schizophrenia [38] were used as the training datasets to calculate individual-level PGS in our sample. Regression analyses were performed to evaluate the prediction of case-control status by individual PGS covarying for the first 5 principal components to allow for possible population stratification. Statistical significance was determined by a permutation-based procedure to correct for the testing of multiple *p*-value thresholds, as implemented by PRSice2. For PGS showing nominally significant prediction (empirical *p* < 0.05 after 10000-replicate permutation test), we further conducted a pathway-based PGS analysis for each of the 7,763 GO BP terms from MsigDB [27, 28] by PRset with a permutation procedure to take pathway size into account [39].

Based on results from the tissue-specific enrichment analysis described above, the cerebellum seems to play a crucial role in ADHD. In order to gain a deeper insights into the cerebellum-related mechanism in ADHD, we further calculated PGS for cerebellum-related brain-imaging phenotypes using UK Biobank GWAS summary statistics [40] to identify cerebellar regions or connections related to ADHD. Thus, 72 structural MRI features and 260 resting-state fMRI features related to the cerebellum were included in this analysis. Individual PGSs were calculated by PRSice2 as above, except that UK Biobank data were used to derive the LD reference panel. Bonferroni correction for testing 332 phenotypes was applied to the permutation *p*-values (significance *p*-value threshold = 0.05/332 = 1.51e^−4^).

Additionally, to evaluate specificity, we computed PGSs using summary statistics from all other available brain imaging GWAS (*N* = 3,713) in UK Biobank and assessed their predictive power for ADHD status in our sample. For each PGS analysis, empirical *p*-values were generated through 10,000 permutation tests. We aggregated and compared empirical *p*-values derived from cerebellum-related PGS analyses (*N* = 222) with those from other brain regions.

### Identifying overlapping risk loci and genes

ADHD risk loci and genes that overlap with other phenotypes were identified by two analyses: (1) Phenome-Wide Association Studies (PheWAS) for the suggestive GWAS signals in the current study for associations with a wide range of other traits. Top-lead SNPs and their high LD proxies (r^2^ > 0.8) were used to search the GWAS Catalog [41] and PhenoScanner v2 [42] for associations (*p* < 5e^−8^); (2) For psychiatric or brain-imaging traits whose PGS models significantly predicted case-control status in our ADHD sample, we conducted variant-to-gene mapping analyses (as described above) on their GWAS summary statistics to identify risk variants and mapped candidate genes for these psychiatric or brain-imaging traits in FUMA [29]. Genes overlapping with the detected ADHD candidate risk genes were further analyzed for their tissue-specific expression profiles and biological functions.

We estimated the correlation in genetic effects between the large European ancestry ADHD GWAS (the latest from PGC ADHD) and the Chinese ADHD sample in the current study using Popcorn, which is designed to manage trans-ethnic scenarios [43]. We also conducted genetic correlation analyses between ADHD and the psychiatric or brain-imaging phenotypes whose PGS models predict ADHD, using GWAS summary statistics from European samples and LD score regression [44].

### Association testing of low frequency/rare variants

Gene-based and gene set-based association analyses on our ADHD sample were carried out using the SKAT test [45] implemented in RVTESTS [46]. We first performed functional annotation for all low frequency/rare variants (MAF < 0.05) using Ensembl Variant Effect Predictor (VEP) [24]. Then, we extracted the low-frequency / rare variants predicted to have high or medium damaging effects, estimated by Ensembl VEP (https://asia.ensembl.org/info/genome/variation/prediction/predicted_data.html) [24], on protein function and identified their canonical transcripts and annotated genes. We further confirmed the pathogenic effect of the included variants with different criteria [25, 47–51]. All genes with > 2 damaging low-frequency / rare variants across ADHD and control samples were included for gene-based association testing. A total of 4,800 genes were examined. For the gene set-based association test, we defined gene sets according to GO BP terms from MsigDB [27, 28] (*N* = 7763). Five genetic principal components were used as covariates in both gene-based and geneset-based tests. Since the genes/genesets are correlated, we performed a 10,000-time permutation test implemented in the program for testing significance, which preserved the family-wise error rate (FWER) without over-correction [52], and *p*_FWER_ < 1e^−3^ was regarded as significant.

## Results

There were 279 ADHD cases and 432 controls, consisting of 196 unrelated controls and 236 pseudo-controls, included in the analyses. The demographic characteristics of ADHD cases and unrelated controls can be found in Supplementary Table 1.

### Genetic association analyses and correlations with gene expression

**Fig. 2:**
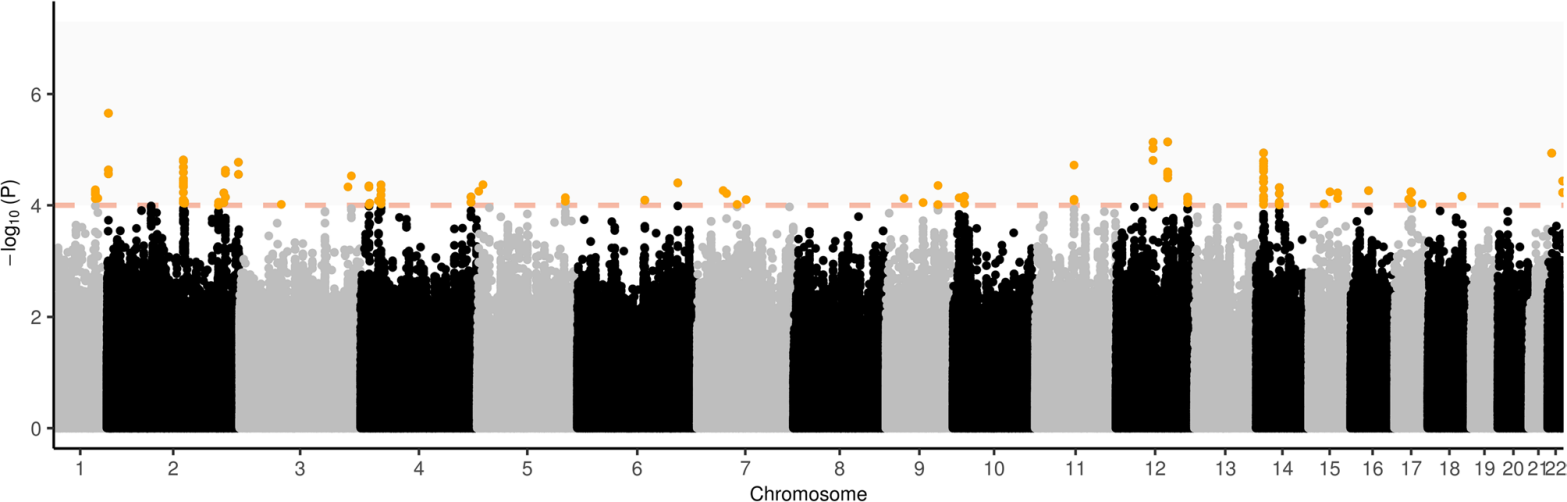
Manhattan plot of GWAS of 279 ADHD cases and 432 controls from Hong Kong samples. Orange line shows the suggestive threshold (*p* = 1e^−4^) adopted in the current study. Identified genomic loci are highlighted in orange (*N* = 41)

The GWAS analysis (lambda = 1.024) identified 48 independent lead variants (r^2^ < 0.1) located in 41 genomic loci at the suggestive threshold (*p* < 1e^−4^) (see Fig. 2; Table 1 and Table S2). None of them reached the genome-wide significance threshold (*p* < 5e^−8^). Gene-based analysis did not show a significant association after multiple testing adjustments. Of the previously identified candidate genes [13], the most significant was *KCTD16* (*p* = 0.0086), which did not survive Bonferroni adjustment for 21 tests (Table S3). There was no significant result from the gene-set based association analyses after multiple testing adjustments.

**Table 1:** Results for the 41 index variants identified at the suggestive threshold in the GWAS of 279 ADHD cases and 432 controls

Locus	Chr	BP (hg19)	rsID	Eff/Non-eff	MAF	OR	SE	*P* value	NearestGene
1	1	208,968,342	rs17013389	A/C	0.199	0.540	0.152	5.27E-05	*RP11-459K23.2*
2	1	218,106,816	rs6667062	C/G	0.431	1.578	0.115	7.48E-05	*LINC00210*
3	2	1,623,553	rs72763534	A/G	0.200	1.994	0.146	2.21E-06	*AC144450.1*
4	2	139,917,014	rs12613166	T/A	0.334	0.594	0.120	1.53E-05	*AC062021.1*
5	2	141,478,468	rs436323	T/C	0.058	2.651	0.248	8.59E-05	*LRP1B*
6	2	207,843,712	rs1006389	C/T	0.099	0.456	0.202	9.92E-05	*CPO*
7	2	218,862,241	rs1001098	T/C	0.431	1.541	0.110	8.98E-05	*TNS1*
8	2	221,965,082	rs6739103	A/G	0.272	0.560	0.137	2.35E-05	*AC011233.2*
9	2	241,754,034	rs73012754	A/G	0.259	1.736	0.128	1.68E-05	*KIF1A*
10	3	64,173,901	rs1035275	C/T	0.236	0.580	0.140	9.65E-05	*PRICKLE2:PRICKLE2-AS3*
11	3	184,176,114	rs9854433	C/A	0.495	1.597	0.112	2.96E-05	*EIF2B5*
12	4	12,889,878	rs223917	C/T	0.205	1.841	0.150	4.49E-05	*RP11-22A3.2*
13	4	14,744,974	rs10010400	C/T	0.071	2.601	0.244	9.20E-05	*LINC00504*
14	4	28,040,589	rs6840350	A/G	0.190	0.525	0.164	8.28E-05	*AC007106.1*
15	4	31,290,243	rs60139026	G/T	0.453	1.588	0.113	4.29E-05	*RP11-315A17.1*
16	4	183,085,906	rs11935992	G/A	0.119	0.432	0.211	7.06E-05	*TENM3:RP11-402C9.1*
17	5	159,955,315	rs1895317	T/C	0.207	0.561	0.146	7.28E-05	*ATP10B*
18	6	90,023,021	rs79488521	A/T	0.385	0.631	0.117	8.08E-05	*GABRR2*
19	6	144,268,094	rs6930726	A/C	0.314	1.649	0.122	3.96E-05	*PLAGL1*
20	7	37,427,783	rs60704414	G/A	0.326	1.660	0.126	6.14E-05	*ELMO1*
21	7	53,186,040	rs6954000	T/G	0.465	0.639	0.115	9.67E-05	*HAUS6P1*
22	7	71,145,041	rs376468499	T/C	0.141	1.793	0.148	7.93E-05	*WBSCR17*
23	9	90,691,910	rs12684075	T/C	0.491	1.546	0.111	8.95E-05	*RP11-350E12.4*
24	9	112,592,855	rs7867595	A/G	0.140	1.866	0.153	4.41E-05	*PALM2:PALM2-AKAP2:AKAP2*
25	10	8,109,576	rs3847417	T/G	0.282	1.594	0.118	7.34E-05	*GATA3*
26	10	15,117,423	rs12219813	A/G	0.359	0.619	0.120	6.94E-05	*DCLREQCP1*
27	11	70,405,685	rs7127137	C/T	0.496	0.642	0.112	7.82E-05	*SHANK2*
28	12	62,006,325	rs4758832	T/C	0.184	0.516	0.148	7.33E-06	*RP11-410B16.1*
29	12	90,095,173	rs11105379	C/T	0.210	1.853	0.137	7.26E-06	*ATP2B1*
30	12	124,361,292	rs56044291	T/C	0.054	2.741	0.254	7.15E-05	*DNAH10*
31	14	33,554,821	rs6571581	A/C	0.497	0.613	0.112	1.15E-05	*NPAS3*
32	14	58,228,104	rs1950997	A/G	0.442	0.615	0.120	4.79E-05	*SLC35F4*
33	15	55,136,295	rs1915202	C/T	0.194	1.635	0.126	9.44E-05	*RP11-548M13.1*
34	15	81,166,740	rs28384338	A/G	0.394	1.566	0.112	5.98E-05	*KIAA1199*
35	16	49,946,670	rs6500265	T/C	0.217	1.686	0.129	5.46E-05	*RP11-305A4.3*
36	17	34,350,579	rs7208039	T/C	0.162	0.518	0.167	7.77E-05	*CCL23*
37	17	40,582,296	rs1905339	C/T	0.350	0.618	0.119	5.70E-05	*PTRF*
38	17	43,313,404	rs9891552	A/T	0.063	2.589	0.237	5.92E-05	*FMNL1*
39	18	65,038,142	rs12605748	G/T	0.187	1.808	0.149	6.93E-05	*RP11-563H6.1*
40	22	26,915,245	rs8135081	A/G	0.171	1.996	0.158	1.16E-05	*CTA-445C9.15*
41	22	49,817,197	rs7287788	A/G	0.119	0.453	0.192	3.67E-05	*C22orf34*

Gene-based association test statistics for protein-coding genes showed a nominally significant correlation with gene expression levels in the caudate nucleus (beta = 0.015, SE = 0.0083, *p* = 0.036) and the nucleus accumbens (beta = 0.025, SE = 0.0080, *p* = 0.037) of the basal ganglia. Cell-type-specific gene-expression levels did not show a significant correlation with ADHD gene-based association results.

The location [chromosome (Chr.) position (bp)] maps in hg19. Eff/Non-eff shows the effective and non-effective alleles. MAF shows frequency of effect allele in cases and controls. OR of the effect with respect to effect allele. NearestGene, the gene nearest the SNP based on ANNOVAR annotations implemented by FUMA with default settings.

### Risk variant-to-gene mapping and enrichment analyses

We identified 111 ADHD candidate risk genes (Table S4) from mapping based on the genomic locations, eQTLs and chromatin interactions of the 48 independent lead variants and their high-LD neighboring SNPs. These candidate genes included *POC1B*, which was reported in a recent large-scale PGC ADHD meta-analysis [11], and *SHANK2*, which was described in our previous study [19].

**Fig. 3:**
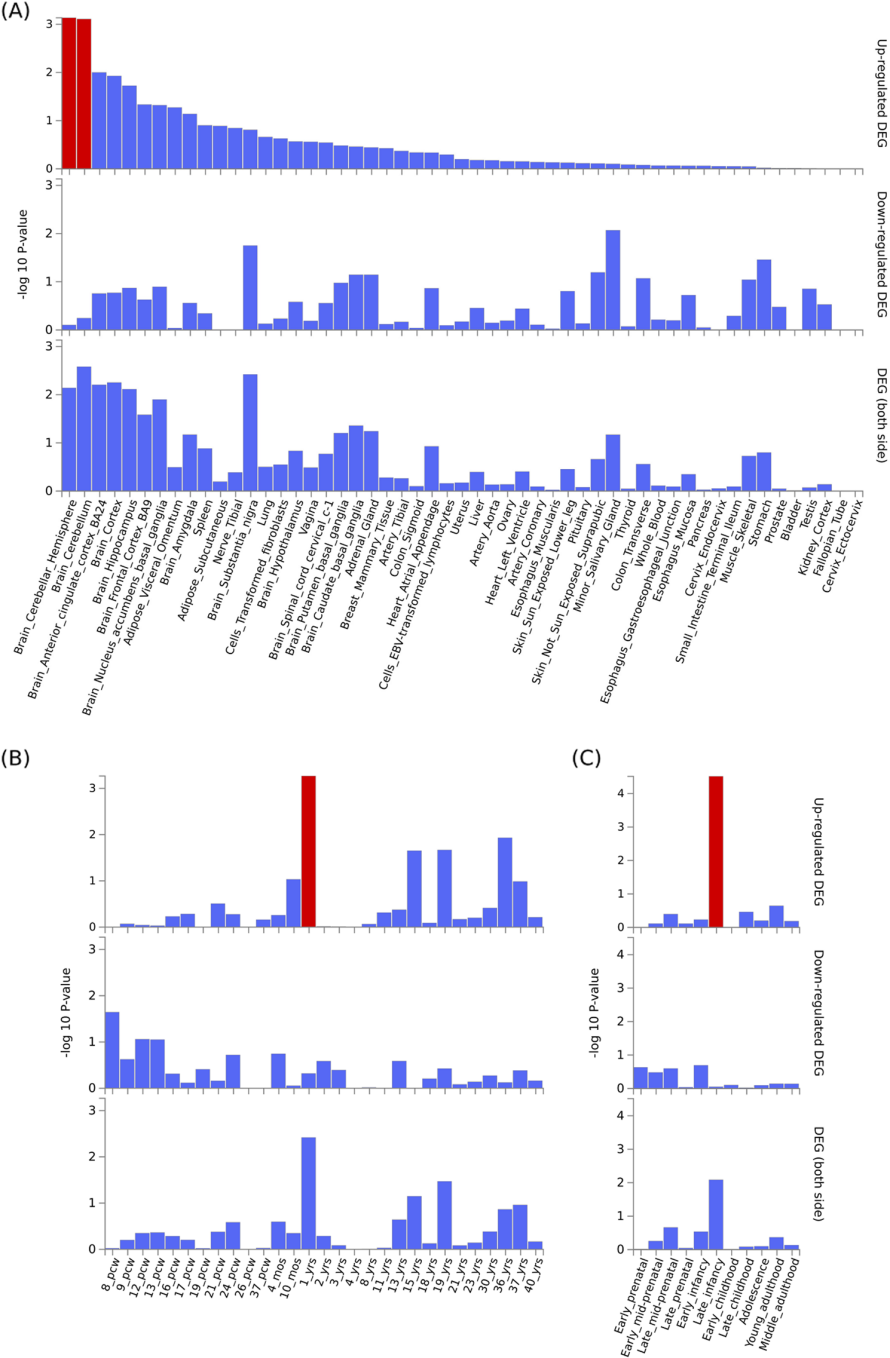
Enrichment in 111 ADHD candidate risk genes among differentially expressed gene (DEG) sets across tissues and in brain tissue. **A** Enrichment among DEGs across 53 specific tissues types available in the GTEx v.7 database. Enrichment was also evaluated among DEGs in brain tissue from Brain Span representing (**B**) 29 different specific ages and (**C**) 11 general developmental stages. DEGs are derived from a differential expression test of each tissue vs. all other tissues for GTEx, and of each age vs. all other ages for BrainSpan data. Highlighted red bars indicate significant enrichment at Bonferroni corrected *p* ≤ 0.05

Bi-clustering analysis of gene expression data (across 111 genes and 53 tissues) revealed a cluster of 16 genes with over expression in brain tissues generally and a smaller cluster of 3 genes with over expression specifically in the cerebellum (Figure S2). In the further enrichment analyses, we found that these 111 candidate risk genes were significantly enriched among up-regulated genes in the brain at 1 year postnatal (*p*_unadj_ = 5.42e^−4^, *p*_adj_ = 0.016) or at the late-infancy development stage, which is defined as between 6 and 12 months postnatal (*p*_unadj_ = 3.06e^−5^, *p*_adj_ = 3.37e^−4^). These genes were also significantly enriched among up-regulated genes in the cerebellar hemisphere (*p*_unadj_ = 7.35e^−4^, *p*_adj_ = 0.039) and cerebellum (*p*_unadj_ = 7.79e^−4^, *p*_adj_ = 0.041), and enriched at a nominally significant level among differentially expressed genes in the substantia nigra (*p*_unadj_ = 0.0038, *p*_adj_ = 0.20) (Fig. 3). They were also highly enriched in a GO cellular components term, presynaptic active zone (*p*_unadj_ = 4.35e^−5^, *p*_adj_ = 0.044); reported genes in GWAS of response to cognitive-behavioral therapy in anxiety disorder (*p*_unadj_ = 2.29e^−6^, *p*_adj_ = 0.0021); and several Reactome immune signaling pathways, including interleukin 9 signaling (*p*_unadj_ = 1.36e^−5^, *p*_adj_ = 0.013), interleukin 21 signaling (*p*_unadj_ = 1.94e^−5^, *p*_adj_ = 0.013), signaling by leptin (*p*_unadj_ = 2.66e^−5^, *p*_adj_ = 0.013), interleukin 15 signaling (*p*_unadj_ = 5.79e^−5^, *p*_adj_ = 0.022) and signaling by cytosolic fgfr1 fusion mutants (*p*_unadj_ = 1.28e^−4^, *p*_adj_ = 0.038) (Figure S3). However, we failed to detect significant enrichment signals in any specific cell type.

### Polygenic score analysis

**Fig. 4:**
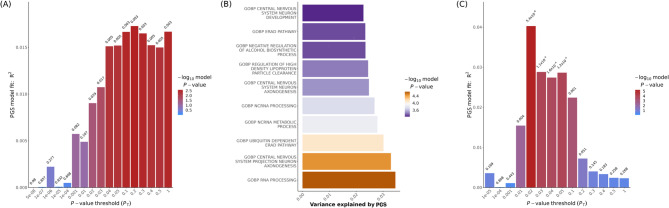
Polygenic score (PGS) for ADHD and brain-imaging phenotype. **A** Bar plot of R^2^ for ADHD prediction across multiple *p*-value thresholds in PGC ADHD summary statistics as the training set. **B** Bar plot shows the enrichment of ADHD signals in different biological pathways. **C** Bar plot of PGS for resting-state fMRI (rs-fMRI) connectivity between [Parietal|Frontal] and [Cerebellum|Temporal] showing the explained variance for ADHD at multiple *p*-value thresholds in the Hong Kong sample

Among the PGSs for five major psychiatric disorders, only the ADHD PGS significantly predicted ADHD case-control status in the current GWAS after permutation tests (*p*-value threshold = 0.2, Lee.R^2^ = 0.012, empirical-*p* = 0.024). Further pathway-based PGS analysis with PGC ADHD summary statistics found that, among all GO terms, “central nervous system projection neuron axonogenesis” achieved the lowest *p*-value after 10,000-replicate set permutations (Lee.R^2^ = 0.027, competitive-*p* = 1.0e^−4^). Among the PGSs for cerebellum-related brain-imaging phenotypes, the PGS for resting-state fMRI (rs-fMRI) connectivity between two components derived from independent component analysis, [Parietal|Frontal] and [Cerebellum|Temporal], which are related to attention/central executive and subcortical-cerebellum networks, had the best performance and passed Bonferroni correction (Lee.R^2^ = 0.027, empirical-*p* = 1.0e^−4^) (Fig. 4 and Figure S4). The empirical *p*-value distribution for non-cerebellum-related brain measures deviated from the expected uniform null distribution, showing decreasing frequencies in smaller *p*-value bins. It suggests that the permutation-based multiple testing adjustment yields more conservative *p*-values. While cerebellum-related measures exhibited similar overall trends, greater variability was observed due to reduced number of observations (fewer included cerebellar measures). Importantly, the proportion of nominally significant associations (*p* < 0.05) among cerebellar measures exceeded the 5% expectation, providing evidence for polygenic associations between specific cerebellar traits and ADHD status (Figure S5).

### Genetic overlap of ADHD with other phenotypes

Among top-lead SNPs and their high LD proxies (r^2^ > 0.8), there were significant associations in GWAS of other phenotypes (details in the Table S5). However, there was no enrichment of these phenotypes in any specific phenotype categories defined in PhenoScanner [42]. We found two shared genes between our 111 ADHD candidate genes and the risk genes indicated by the rs-fMRI connectivity GWAS data, *LRP1B* and *TENM3*, which have an enhanced expression level in the brain and inhibitory neurons [53, 54].

There was no significant trans-ancestry genetic correlation between the European ancestry ADHD GWAS and the current ADHD GWAS, however, our power to detect such correlation is low due to the limited sample size. We did not find a significant genetic association of the European ancestry ADHD with rs-fMRI connectivity between [Parietal|Frontal] and [Cerebellum|Temporal] components.

### Low-frequency / rare variant association test

The gene-based low-frequency / rare variant association test revealed five significant genes, *TEP1* (*p*_FWER_ = 1.58e^−4^), *MTMR10* (*p*_FWER_ = 3.24e^−4^), *TBCC* (*p*_FWER_ = 3.78e^−4^), *DBH* (*p*_FWER_ = 4.31e^−4^), and *ANO1* (*p*_FWER_ = 8.08e^−4^), after 10,000-replicate permutation test (Table 2, Figure S6 and Table S6). In the gene-set-based low-frequency / rare variant analysis with 5860 biological process GO terms, “norepinephrine biosynthetic process” (*p*_FWER_ = 3.30e^−4^) reached significance.

**Table 2:** Variants of each significant genes in low-frequency / rare-variant association analysis

Symbol	uniqID	rsID	Ref	Alt	MAF	MAC (case)	MAC (control)	Consequence	IMPACT
*TBCC*	6:42712809	rs138350475	T	C	5.26E-03	2	5	Missense variant	MODERATE
	6:42712977	rs12175072	C	T	2.71E-02	4	31	Missense variant	MODERATE
*DBH*	9:136508566	rs201600007	C	T	1.49E-03	0	2	Missense variant	MODERATE
	9:136508640	rs13306301	G	A	8.96E-03	10	2	Missense variant	MODERATE
	9:136509403	rs201128036	C	T	2.99E-03	1	3	Missense variant	MODERATE
*ANO1*	11:69933891	rs76718681	G	A	3.21E-02	5	38	Missense variant	MODERATE
*TEP1*	14:20851708	rs117775572	T	C	1.72E-02	11	12	Missense variant	MODERATE
	14:20871973	rs2228035	T	C	2.31E-02	21	10	Missense variant	MODERATE
*MTMR10*	15:31234064	rs6493352	C	T	1.42E-02	14	5	Missense variant	MODERATE

We present minor allele frequency (MAF) and minor allele count (MAC) in cases and controls for alternative allele (Alt)

IMPACT, IMPACT rating estimated by Ensembl VEP [24]

## Discussion

Although our study was underpowered to identify genome-wide significant signals for ADHD, a substantial proportion of the 41 independent risk loci identified as passing the suggestive threshold (*p* < 1e^−4^) implicate genes and neurobiological functions that may play a role in ADHD. These loci implicated 111 candidate risk genes through functional genomic data. The genes were significantly enriched among genes up-regulated in the cerebellum compared to other tissues. The traditional view of the cerebellum is that it plays a critical role in the fine control and coordination of movements to achieve automatic and smooth execution with practice [55, 56]. Growing evidence suggests that the cerebellum is similarly involved in the fine control and coordination of perceptual, cognitive and emotional processes [57–60].The symptoms of ADHD may partly reflect the dysregulation of attention, impulses, emotional responses, and motor activities, resulting from cerebellar dysfunction [61–63]. Consistent with a role of the cerebellum in ADHD, reduced cerebellar volume has been reported in ADHD [64] and to be associated with greater symptom severity [65].

Using polygenic scores, we found ADHD to be genetically correlated with the resting-state functional MRI connectivity between the attention/central executive and subcortical-cerebellum networks. Against the empirical *p*-value distribution of non-cerebellum-related brain measures, our findings highlight the cerebellum as one of the critical nodes in ADHD pathogenesis. Disordered connectivities of the cerebellum with the frontal cortex and basal ganglia have significant influences on attention, working memory, planning, inhibition, and coordinated movements, required for flexible, adaptive behaviors [66, 67]. The disruption of these processes has been proposed as being fundamental in the genesis of ADHD symptoms [68–70]. The overlapping risk genes between ADHD and this functional connectivity are *LRP1B* and *TENM3*. *LRP1B* is a cell-surface receptor which is expressed in the brain and is involved in cell migration and synaptic plasticity [71]. *LRP1B* was associated with motor and cognitive ability in 2-year old [72] and with hyperlocomotion in people using cocaine [73]. *LRP1B* was the only gene found to have altered expression in both ventral and dorsal striatum by repeated administration of cocaine in rats [73]. *TENM3* is a Teneurin involved in guiding cortical neuronal migration and synapse formation [74] in the hippocampus [75], thalamus and striatum [76]. *TENM3* knockout mice were delayed in developing motor skills [76]. Teneurins are genetically associated with bipolar disorder and schizophrenia [77]. *TENM3* is associated with childhood autoimmune diseases [78], and the presence of autoantibodies to N-methyl-d-aspartate-receptor subunit-NR1 (NMDAR1), the most common antigen for anti-brain autoantibodies [79]. These results suggest that some of the identified variants are relevant to ADHD by altering neuronal development and the coordinated functioning of the cerebellum and other brain regions.

The current study provides evidence supporting the importance of the catecholamine system, especially in the cerebellum, in ADHD. We identified the low-frequency / rare-variant associations of ADHD with *DBH* (Dopamine beta-hydroxylase), whose encoded protein converts dopamine to norepinephrine. The identified ADHD candidate genes were nominally enriched among genes expressed in the midbrain dopaminergic regions [80]. The primary pharmacologic effect of psychostimulant drugs for ADHD is to elevate the activity of central dopamine and norepinephrine [81]. Interestingly, several studies have demonstrated the crucial role of the cerebellum in dopamine deficit-related neurological and psychiatric disorders [82–84]. Recent neuroimaging evidence indicates connectivity between cerebellum and midbrain dopaminergic regions (substantia nigra and ventral tegmental area), both structurally [85–88] and functionally, and that this connectivity is altered in Parkinson’s disease [89]. In addition, lesional and axonal tracing experiments on rodents have shown that the midbrain dopaminergic cell groups are the origin of the cerebellar extrinsic dopaminergic fibers [90, 91]. In mouse models, dopamine acts on D2 receptors in parts of the cerebellum to alter the propensity for social novelty and sociability without changing their motor coordination and other functions [92]. A direct efferent influence from the cerebellum to the midbrain dopaminergic nuclei to modulate reward processing and social behavior has also been demonstrated [93]. These studies indicate potential reciprocal, direct connectivities between the cerebellum and the midbrain dopaminergic regions, and the alterations in these functional connectivities may affect cognitive and emotional regulation and social behavior and lead to psychiatric symptoms. Cerebellar function is also modulated by norepinephrinergic input from the locus coeruleus as part of its effects on attention, arousal, and cognition [94, 95].

The significant enrichment of the candidate risk genes up-regulated in late-infancy/1-year brain development indicates that this period may be critical for ADHD vulnerability. This stage is a landmark for human nervous system development with a rapid increase in synaptic density in the cerebral cortex, and a greater rate of growth of the cerebellum relative to the rest of the brain. The ratio of the volume of the cerebellum to the intracranial volume reaches a plateau in late infancy. Thus, any disruptions to neural processes such as synaptic proliferation, formation, and pruning at this stage may have a disproportionate effect on the cerebellum, and subsequently produce symptoms of cerebellar dysfunction present in ADHD [64, 67, 96].

The current study demonstrated, through pathway-enrichment and set-based association analyses, convergent risk of common and low-frequency / rare variants that inform potential biological mechanisms in ADHD. An elevated load of low-frequency / rare, damaging variants in *MTMR10*, *DBH*,* ANO1*,* TEP1*, and *TBCC* was observed in ADHD patients. According to Genecard [97], *MTMR10*, Myotubularin related protein 10, has an enhanced expression level in oligodendrocytes and astrocytes and is part of “Brain - Nervous system development” and “Astrocytes - Nervous system maintenance” annotation clusters. *ANO1*, encodes a protein required for the Ca^2+^-dependent process extension of radial glial cells, especially in the developing brain, and is involved in regulating the excitability of neurons. *TEP1*, Telomerase associated protein 1, belongs to the “B-cells - Humoral immune response” cluster. *TBCC*, Tubulin folding cofactor C, is annotated to “Immune cells - Immune response” and “T-cells - Immune response” expression clusters. These results implicate, in addition to neural processes, the involvement of the immune system in ADHD. There is indeed increasing evidence supporting the association of immune system dysregulation with psychiatric disorders, including schizophrenia [98–100], depression [101], and autism spectrum disorder [102–104]. Previous studies showed that immune processes might regulate the nervous system and have an impact on the elimination and plasticity of synapses during development [105]. It has been proposed that modern lifestyles (e.g., hygiene) might have deprived infants of co-evolved immunoregulatory organisms, with adverse effects on neural development and increasing vulnerability to psychiatric disorders [106].

In the current study, an accumulation of common risk variants for ADHD identified in European ancestry GWAS (measured by a PGS) was found to be significantly associated with case-control status in our Asian ancestry sample. This is consistent with the moderately large estimated SNP-based genetic correlation of 0.39 for ADHD between Europeans and Chinese [13]. *POC1B*, a gene previously found in a genome-wide significant locus of ADHD in the European population [11], was replicated in the current study, potentially implicating a trans-ethnic effect on ADHD. *KCTD16* showed a nominally significant association with ADHD in a previous Chinese ADHD GWAS study [13] and in the current study. These results demonstrate that at least some genetic factors are shared across ancestry groups, supporting the existence of common biological mechanisms underpinning ADHD.

The current study has some limitations. First, the sample size is limited, affecting the statistical power to detect variants with modest effect size and identify individual risk genes. Based on the power calculation, the current sample size provides 80% statistical power to detect common risk variants with an OR greater than 1.85 and 70% power to detect variants with an OR greater than 1.68, assuming a significance level of 5e^−8^ and a MAF of > 0.05. Second, all participants are male, which may affect the generalization of the results. Nevertheless, these current findings likely foreshadow the identification of individual risk genes in larger East Asian cohorts or meta analyses.

## Conclusion

As far as we know, this is the first GWAS study using a Hong Kong ADHD sample. The current study identified convergent risk factors from common and low-frequency / rare variants, which implicates vulnerability in late-infancy brain development, affecting especially the cerebellum, and the involvement of immune processes. Furthermore, our findings show that diverse ancestry groups share some genetic factors driving ADHD.

## Supplementary Information

Supplementary Material 1: Table S1. Demographic characteristics of cases and unrelated samples. Table S2. Results for the 41 identified index variants in the current GWAS and PGC ADHD. Table S3. Results from candidate gene-based association analysis by MAGMA. Table S4. Genes mapped to independent risk loci in FUMA. Table S5. Associations of suggestive ADHD GWAS signals with other traits from PhenoScanner. Table S6. Functional annotations of tested low-frequency / rare variants in association analysis.

Supplementary Material 2: Figure S1. Multidimensional scaling (MDS) plots. Figure S2. Heatmap for gene expression of 111 ADHD candidate risk genes across 53 specific tissues types available in the GTEx v.7 database. Figure S3. Bar chart for pre-defined gene sets with overrepresentation of 111 ADHD. Figure S4. Distribution plot of PGS in cases versus controls. Figure S5. Permutation-based *p*-value distribution of cerebellum-specific and other brain region PGS associations with ADHD. Figure S6. Distribution plot of MAF of variants in our dataset against their corresponding MAF in the 1KG EAS panel. Appendix S1. eQTL datasets in FUMA used for gene-mapping. Appendix S2. Chromatin interaction datasets in FUMA used for gene-mapping.

## Data Availability

The generated summary statistics and datasets analyzed during the current study are available from the corresponding author upon reasonable request.

## Abbreviations

ADHD: Attention-deficit/hyperactivity disorder
GWAS: Genome-wide association study
PGS: Polygenic score
PGC: Psychiatric Genomics Consortium
FUMA: Functional Mapping and Annotation
SNP: Single nucleotide polymorphism
