# Supplementary material for: Common and rare variant analyses implicate late-infancy cerebellar development and immune genes in ADHD
Source: J Neurodev Disord. 2025 Jun 20;17:34. doi: 10.1186/s11689-025-09626-4 (PMC12180188; doi:10.1186/s11689-025-09626-4)
Supplement: Supplementary file 2 — Supplementary Material 2: Figure S1. Multidimensional scaling (MDS) plots. Figure S2. Heatmap for gene expression of 111 ADHD candidate risk genes across 53 specific tissues types available in the GTEx v.7 database. Figure S3. Bar chart for pre-defined gene sets with overrepresentation of 111 ADHD. Figure S4. Distribution plot of PGS in cases versus controls. Figure S5. Permutation-based p-value distribution of cerebellum-specific and other brain region PGS associations with ADHD. Figure S6. Distribution plot of MAF of variants in our dataset against their corresponding MAF in the 1KG EAS panel. Appendix S1. eQTL datasets in FUMA used for gene-mapping. Appendix S2. Chromatin interaction datasets in FUMA used for gene-mapping. [file 11689_2025_9626_MOESM2_ESM.docx]

**Supplementary Materials**

**
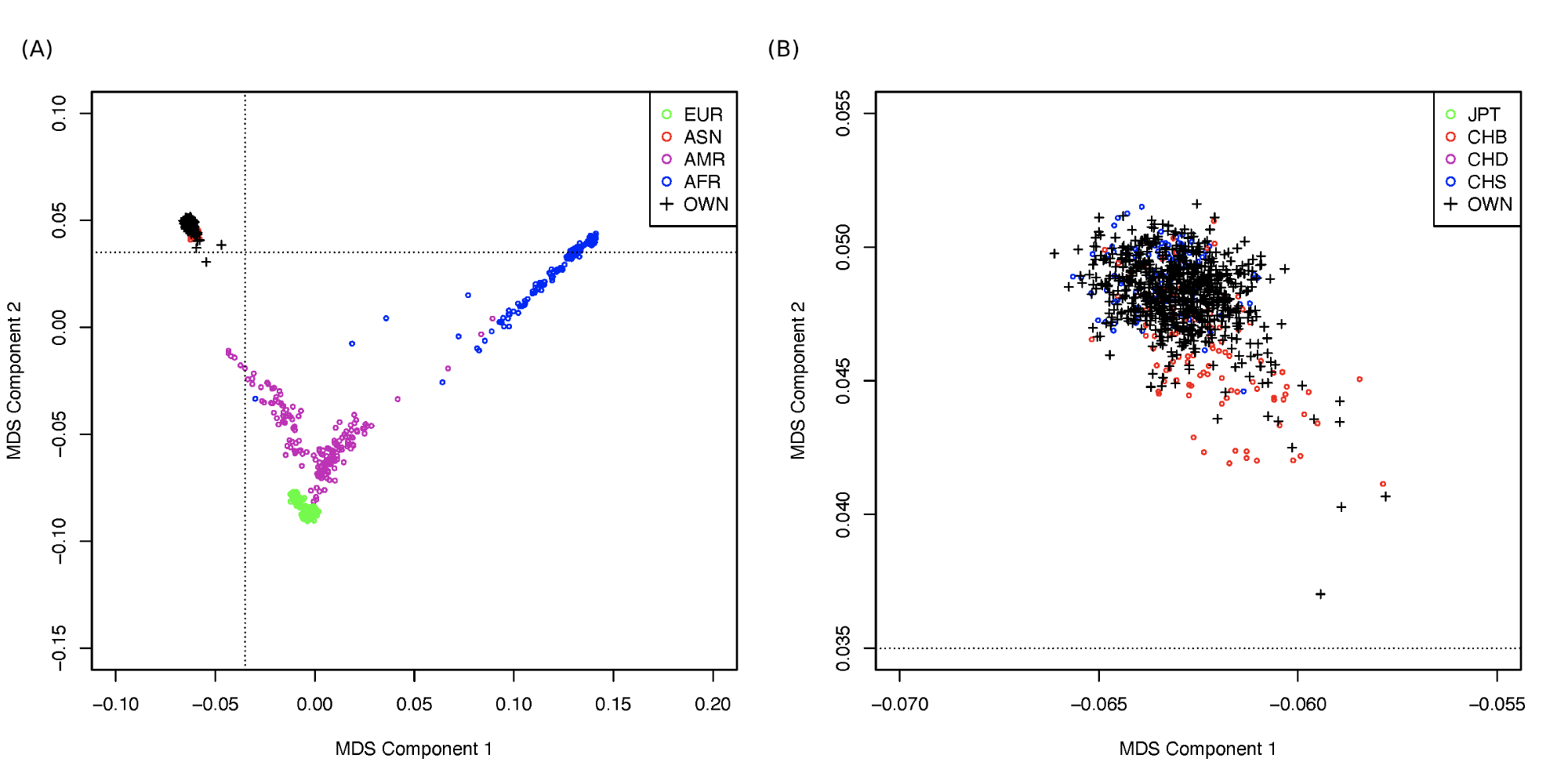
**

**Figure S1.** Multidimensional scaling (MDS) plots of the first two principal components between individual samples of (A) the Hong Kong samples (OWN) and 1000 Genomes Project (EUR, European; ASN, East Asian; AMR, Admixed American; AFR, African); (B) the Hong Kong samples (OWN) and East Asian population from 1000 Genomes Project (JPT, Japanese in Tokyo, Japan; CHB, Han Chinese in Beijing, China; CHD, Chinese in Denver, Colorado ; CHS, Southern Han Chinese).


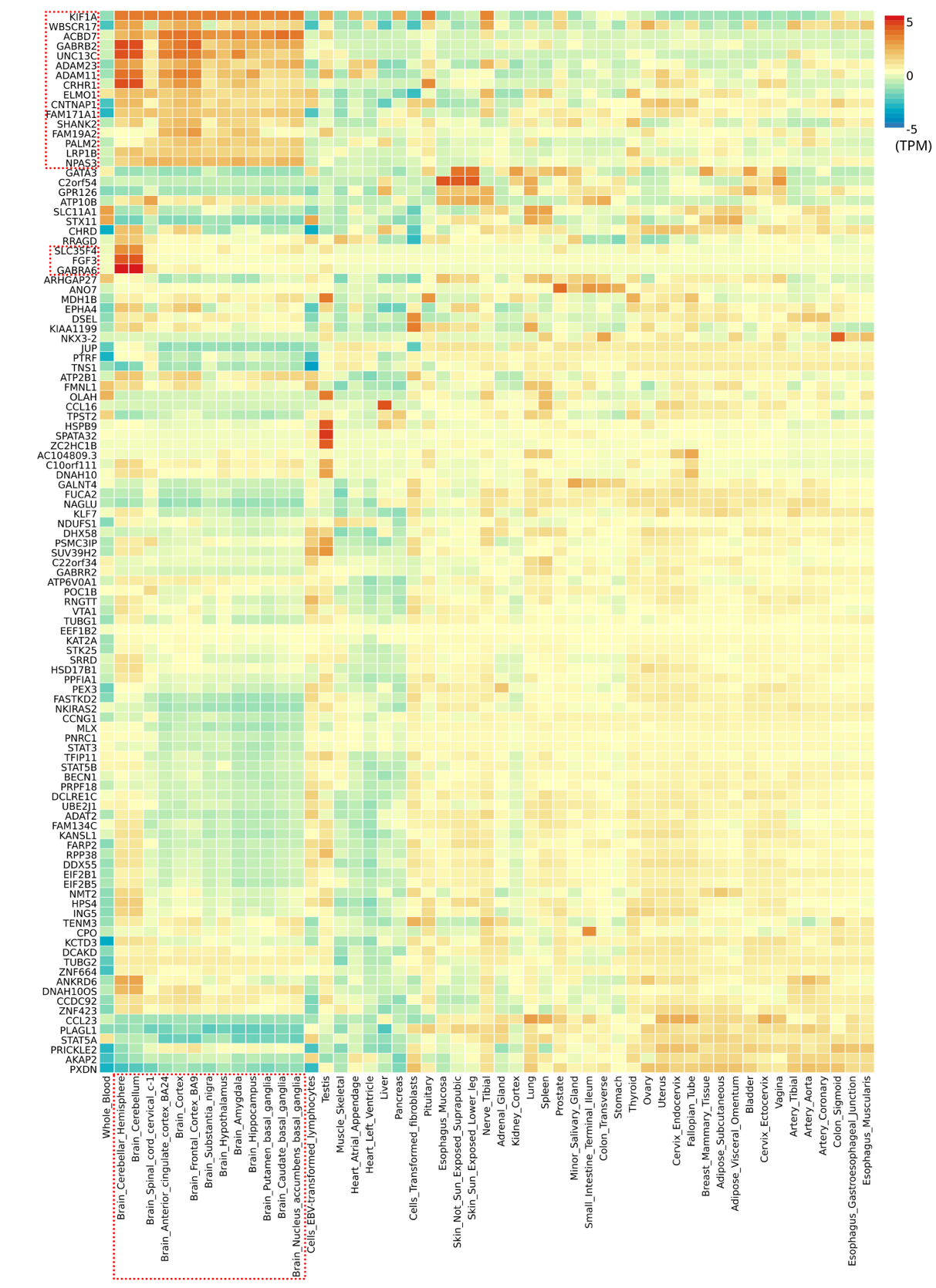


**Figure S2.** Heatmap for gene expression of 111 ADHD candidate risk genes across 53 specific tissues types available in the GTEx v.7 database. Gene expression values are measured as TPM (Transcripts Per Million). Both tissues (columns) and genes (rows) have been ordered by hierarchical clustering implemented in FUMA. A cluster of 16 genes with over expression in brain tissues generally and a smaller cluster of 3 genes with over expression specifically in the cerebellum are observed.


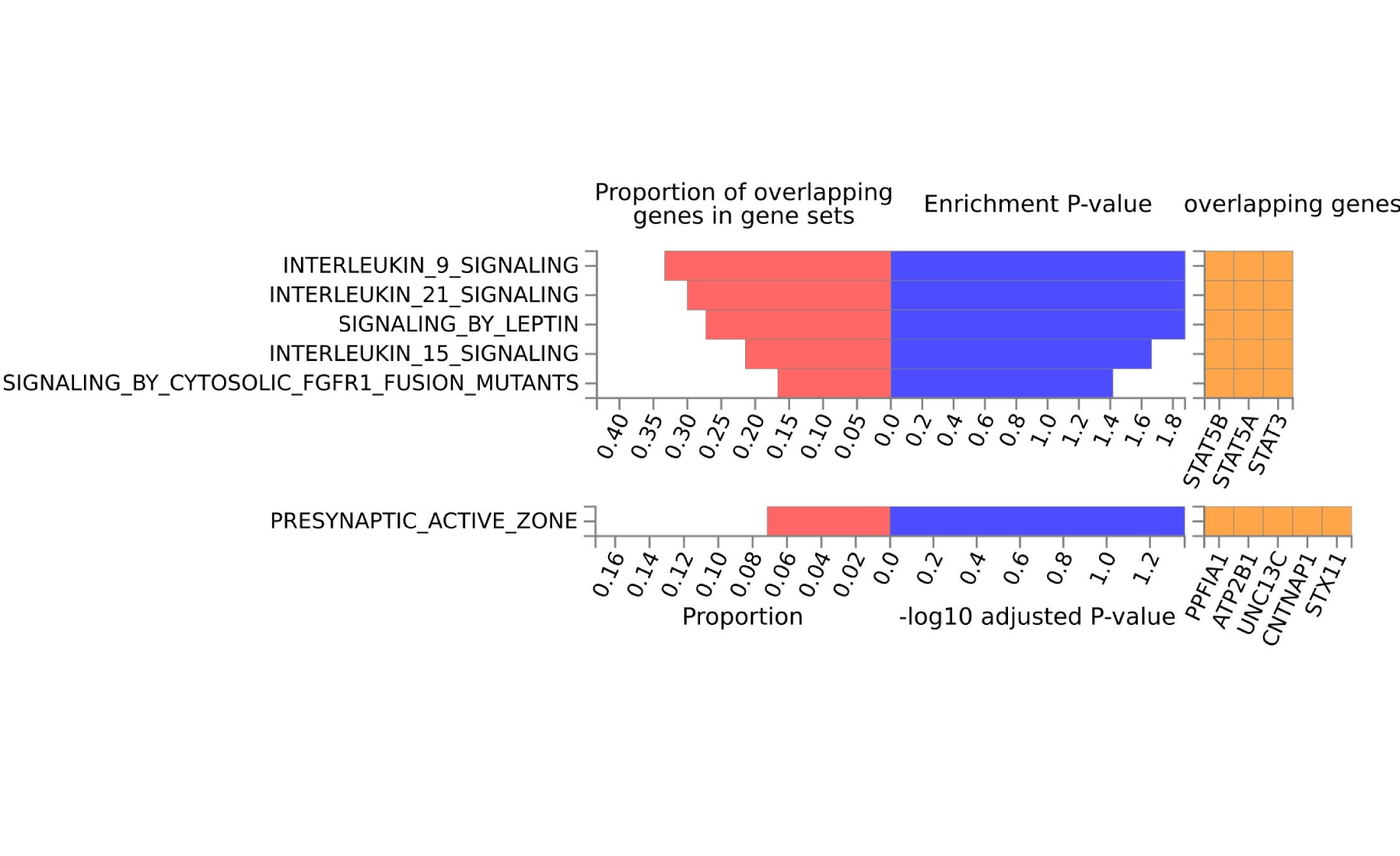


**Figure S3**. Overrepresentation of 111 ADHD candidate risk genes in pre-defined gene sets from WikiPathways and GO cellular components in MSigDB. Multiple testing correction is performed per category (i.e., WikiPathways and GO cellular components in MSigDB). Only significant gene sets (with adjusted *p*-value < 0.05, or -log_10_*p* > 1.30) are shown.

(B)

(A)


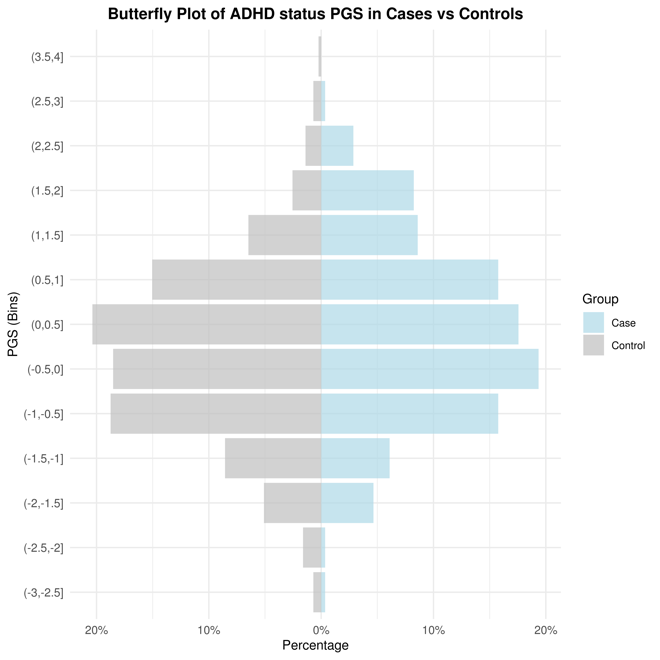

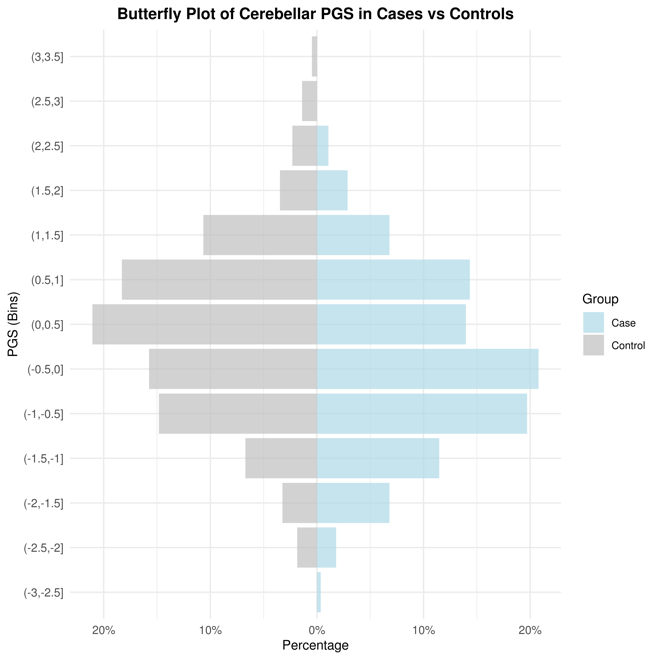


**Figure S4.** Distribution plot of PGS in cases versus controls. (A) PGS predicting ADHD status in HK samples, using PGC ADHD summary statistics as the training set. (B) PGS predicting ADHD status in HK samples, using the summary statistics of resting-state fMRI (rs-fMRI) connectivity between [Parietal|Frontal] and [Cerebellum|Temporal]. PGS, polygenic score.


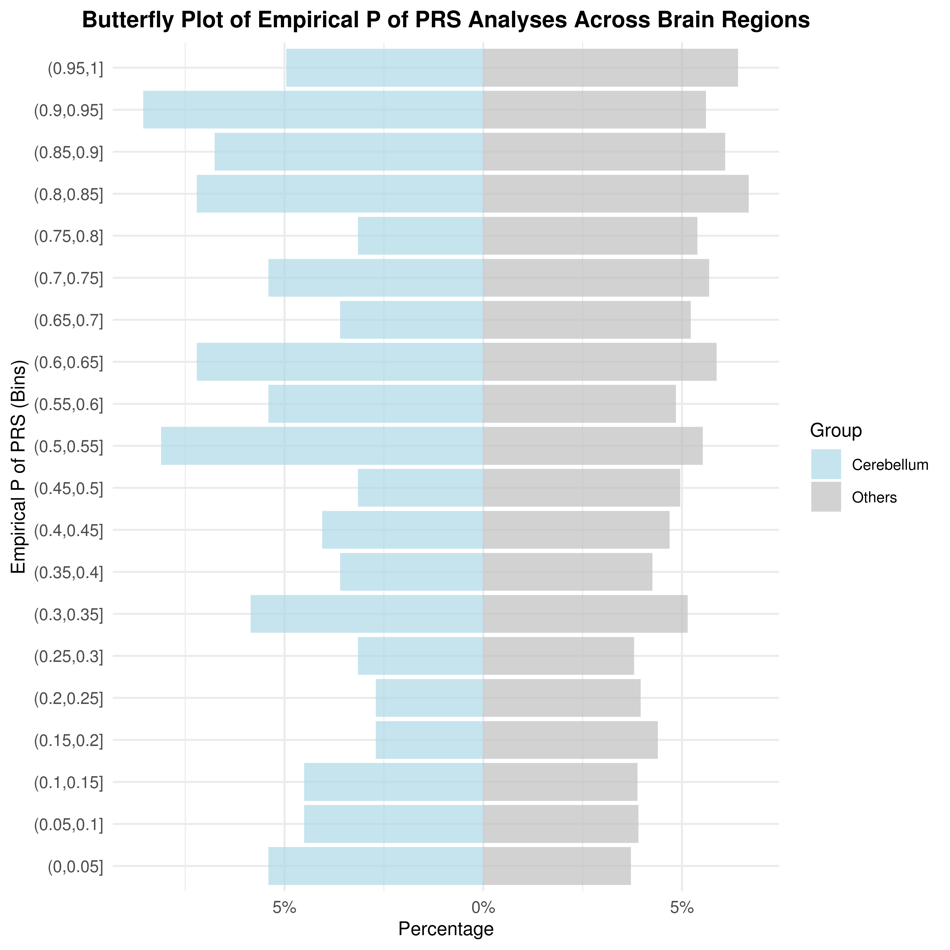


**Figure S5**. Permutation-based p-value distribution of cerebellum-specific and other brain region PGS associations with ADHD. Polygenic scores (PGS) derived from brain imaging summary statistics (total N = 3,935) were tested for ADHD prediction. Empirical p-values were generated through 10,000 permutations per PGS analysis. The plot contrasts cerebellum-related PGS (N = 222, left wing) against non-cerebellar brain region PGS (right wing).


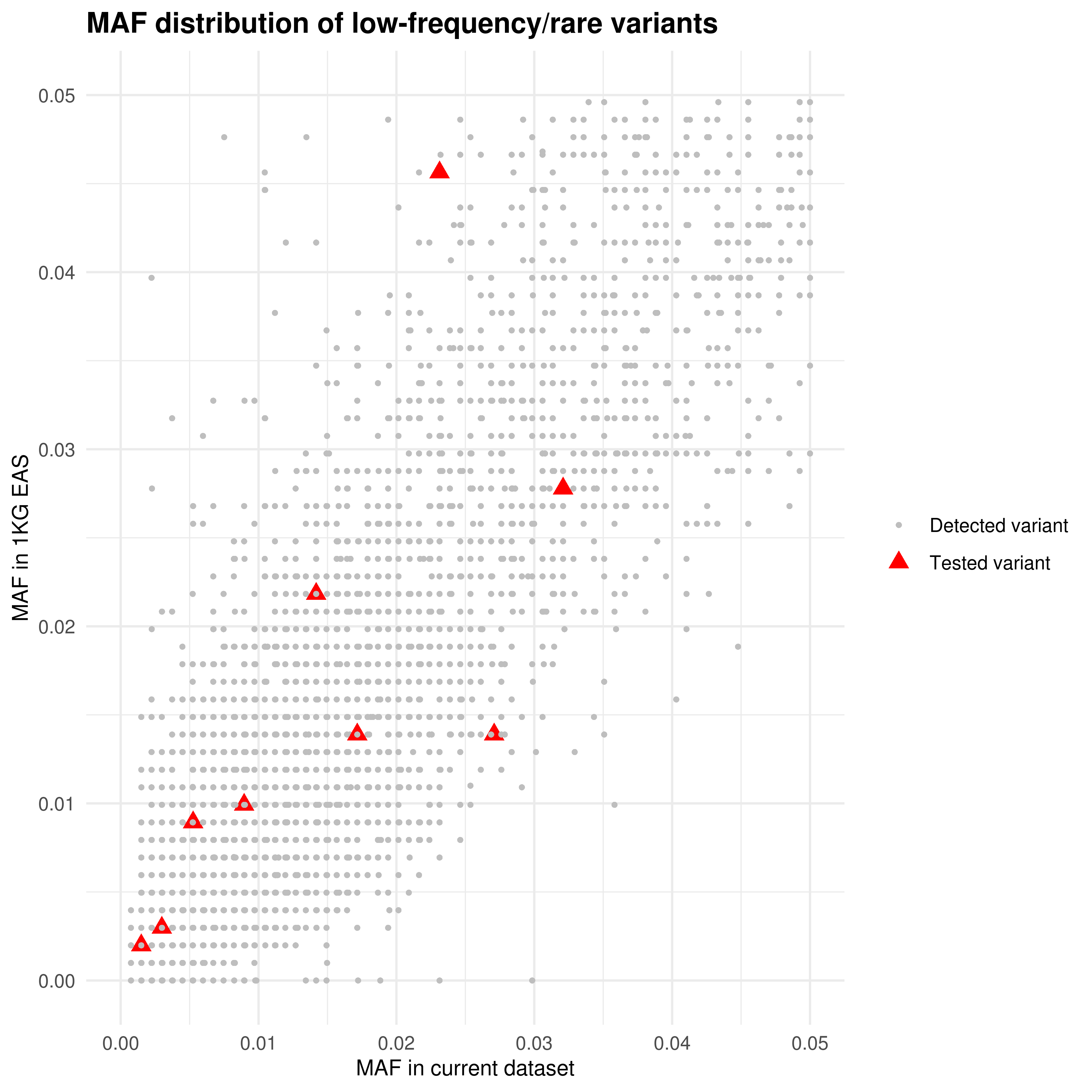


**Figure S6**. Distribution plot of MAF of variants in our dataset against their corresponding MAF in the 1KG EAS panel.

**Appendix S1. eQTL datasets in FUMA used for gene-mapping**

eQTLcatalogue/BrainSeq_ge_brain.txt.gz

eQTLcatalogue/Lepik_2017_ge_blood.txt.gz

eQTLcatalogue/Schwartzentruber_2018_ge_sensory_neuron.txt.gz

eQTLcatalogue/TwinsUK_ge_blood.txt.gz

PsychENCODE/PsychENCODE_eQTLs.txt.gz

scRNA_eQTLs/PBMC.txt.gz

eQTLGen/eQTLGen_cis_eQTLs.txt.gz

BloodeQTL/BloodeQTL.txt.gz

CMC/CMC_SVA_cis.txt.gz

CMC/CMC_SVA_trans.txt.gz

CMC/CMC_NoSVA_cis.txt.gz

CMC/CMC_NoSVA_trans.txt.gz

BRAINEAC/CRBL.txt.gz

BRAINEAC/FCTX.txt.gz

BRAINEAC/HIPP.txt.gz

BRAINEAC/MEDU.txt.gz

BRAINEAC/OCTX.txt.gz

BRAINEAC/PUTM.txt.gz

BRAINEAC/SNIG.txt.gz

BRAINEAC/TCTX.txt.gz

BRAINEAC/THAL.txt.gz

BRAINEAC/WHMT.txt.gz

BRAINEAC/aveALL.txt.gz

GTEx/v8/Brain_Amygdala.txt.gz

GTEx/v8/Brain_Anterior_cingulate_cortex_BA24.txt.gz

GTEx/v8/Brain_Caudate_basal_ganglia.txt.gz

GTEx/v8/Brain_Cerebellar_Hemisphere.txt.gz

GTEx/v8/Brain_Cerebellum.txt.gz

GTEx/v8/Brain_Cortex.txt.gz

GTEx/v8/Brain_Frontal_Cortex_BA9.txt.gz

GTEx/v8/Brain_Hippocampus.txt.gz

GTEx/v8/Brain_Hypothalamus.txt.gz

GTEx/v8/Brain_Nucleus_accumbens_basal_ganglia.txt.gz

GTEx/v8/Brain_Putamen_basal_ganglia.txt.gz

GTEx/v8/Brain_Spinal_cord_cervical_c-1.txt.gz

GTEx/v8/Brain_Substantia_nigra.txt.gz

**Appendix S2. Chromatin interaction datasets in FUMA used for gene-mapping**

EP/PsychENCODE/EP_links_oneway.txt.gz

HiC/Giusti-Rodriguez_et_al_2019/Adult_Cortex.txt.gz

HiC/Giusti-Rodriguez_et_al_2019/Fetal_Cortex.txt.gz

HiC/GSE87112/Dorsolateral_Prefrontal_Cortex.txt.gz

HiC/GSE87112/Hippocampus.txt.gz

HiC/GSE87112/Neural_Progenitor_Cell.txt.gz
